# Feeling low and unhappy together? An actor-partner-interdependence model uncovering the linkage between different operationalizations of relationship quality and depression in different-sex couples

**DOI:** 10.1371/journal.pone.0274756

**Published:** 2022-11-16

**Authors:** Corina Aguilar-Raab, Friederike Winter, Marc N. Jarczok, Beate Ditzen, Marco Warth

**Affiliations:** 1 Institute for Medical Psychology, Medical Faculty, University Heidelberg, Heidelberg, Germany; 2 Department of Psychosomatic Medicine and Psychotherapy, University Clinic Ulm, Ulm, Germany; PhD, PLOS, UNITED KINGDOM

## Abstract

Some studies suggest a bi-directional association between low relationship quality and depression. However, the social impact of depression and the potential preventative effects of healthy relationships are not yet sufficiently understood, as studies have shown heterogenous results for effects in both directions. Therefore, the main aim of this study was to differentiate the actor and partner effects of this association more comprehensively using two measures to capture characteristics of relationship quality–firstly regarding general aspects of social system quality and secondly considering specific aspects of the romantic relationship. 110 different-sex couples were included, being separated in partners with highly pronounced depressive symptoms in women (Cw/DW) versus partners with low depressive symptoms (LDCs). We investigated effects cross-sectionally using multi-group analyses to predict relationship (couple specific questionnaire: PFB) versus system quality (general system quality questionnaire: EVOS) in a step-by-step approach, modelling actor and partner effects with variation within and across both groups and then comparing the results to models with equal actor and partner effects. Depression was measured with the PHQ-9. With regard to the relationship between depressive symptoms and system quality, the model that constrained actor and partner effects to be equal across both groups was preferred and showed negative significant actor effects across gender and groups. Concerning the association between depressive symptoms and relationship quality, the model constraining actor and partner effects to be equal within groups had the best fit to the data and revealed a negative partner effect in LDCs.

Conclusions

Controlling for the moderating variable of clinically relevant depressive symptoms, we found evidence for actor and partner effects, which differed between the two relationship measures. This underlines the importance to reflect how relationship quality is operationalized. The negative partner effect on relationship quality in LDCs emphasizes that even in a non-clinical context, depressive symptoms negatively impact the perceived relationship quality of both women and men. This suggests that addressing the relationship is important in non-clinical preventive contexts and calls for integrating the partner into counselling or trainings.

## Introduction

The majority of people live in romantic relationships most of their adult life [1]. Close and positive social relationships serve as an important, health-promoting resource [2]. For instance, emotional self-disclosure or social reinforcement through praise in verbal and non-verbal communication can act as protection against susceptibility to mental and physical illness [3]. A high level of social integration and perceived relationship quality has been found to act as a buffer against mortality [4]. Positive relationships can serve as a basis of emotional security and increase quality of life and life satisfaction [5–7]. Moreover, healthy relationships can be a major source of support in challenging and destabilizing situations and serve as a buffer against short-term and chronic stressors [8].

What if there is a lack of relationship quality and presence of psychopathology? Maintaining a positive, nurturing relationship is a challenge for many–especially as the relationship increases in duration: Beach’s marital discord model suggests that distress between partners leads to depressive symptoms due to an increase in stress and hostility as well as a reduction of support [9, 10]. The model states that stress increases due to relationship behaviors such as verbal and physical aggression or threats of separation while relationship support is theorized to decrease due to factors such as reduced couple cohesion or intimacy [10]. It is considered scientifically proven that marital discord and relationship distress are significant precursors to the onset of a depressive episode since in a several studies, marital dissatisfaction has been shown to predict an increase in depressive symptoms over time [11–13]. This is relevant because depression is a highly prevalent disorder [14]. In Germany, more than ten percent of individuals were diagnosed with a major depressive disorder in 2017 [15]. Prevalences have been reported to increase particularly in men, indicating the gender difference to diminish. However, women still experience depressive disorders almost twice as often as men [15]. The gender gap has been reported to emerge early in childhood at age 12, then to narrow and remain stable in adulthood [16]. Factors explaining the gender difference in MDD are likely to be intersected. One hypothesis to explain the gender gap in depression are traditional masculine norms [17], that prevent men from seeking psychological treatment [18], thus resulting in an underestimation of depression in men.

But even on subclinical levels of depression, individuals can suffer from depressive symptoms and lead to higher risk for future MDD [19, 20]. Hence, it is important to include both high levels of depressive symptoms and subclinical levels of depression when studying the association with relationship and system quality.

The experience of a depressive disorder in one partner can lead to a burden in the other partner, which is negatively associated with relationship quality and increases their vulnerability for depressive symptoms [21]. Factors associated with satisfied and long-lasting relationships are addressed in the vulnerability–stress–adaptation model of marital development of Karney and Bradbury [22] which combines individual and dyadic factors: Not only partner strength and vulnerabilities, but also distressing events as well as couples’ adaptive interactions and coping mechanisms are important to consider in different levels of couple functioning [23, 24]. The reciprocal nature of individual distress and aspects of relationship quality or relationship satisfaction seems obvious in light of this, and has also been confirmed in a number of studies [25, 26]. Depressed individuals report higher interpersonal distress and perceive their relationship as less satisfying [27]. Individuals with depressive symptoms might withdraw more often or seek reassurance and support, although they interact and communicate less positively [28]. They can be less responsive to communicate with their partner or appreciate their efforts. Dissatisfaction with the relationship may increase, the burden on both can further grow, the non-depressed partner’s initial support may decrease over time, while the implicit or explicit rejection can become more and more obvious. Hence, a vicious circle can begin. Negative emotional contagion and the dynamics of emotional synchrony can add to this vicious cycle and may end up in a decrease in relationship satisfaction [29]. The stress level can rise and require adaptation and coping strategies, which, however, may become increasingly less tangible. The above-mentioned aspects of emotional and also sexual intimacy and closeness can deteriorate, the commitment to each other might be questioned. A negative communication style characterized by hostility can build up with mutual accusations and blame. The skills necessary for a functioning relationship may not be present or available due to the pathological impairment and the negative interaction circle. Beyond emotional and cognitive aspects such as sadness and rumination, the social skills-domain in depression is also unfavorably affected [30]. Some findings indicate a lower capacity for empathy and perspective taking [31–33]. Expecting hostile behavior from others, they can fear receiving less support and yet ask for more reassurance [34]. The self- and other perception and the communication of needs as well as the emotional (co-)regulation can be not particularly functional and contributes to aggravation and chronification.

Complex interpersonal effects as outlined above can be described and explained with the interdependence theory [35]. Within the framework of this theory, it is suggested that decisions and behavior are essentially–not as usually focused solely based on intrapersonal traits–but primarily on interpersonal processes. According to Kelley and Thibaut, social interactions are a function of situational factors, of traits and behaviors of the individual interacting persons partner A and partner B embedded in their interaction behavior. The theory specifies that decisions and behavior are always to be understood in a situational context. This context or structure can be characterized by at least six features such as the mutuality of dependence, which means that according to the degree of dependence of A on B, it leads to more or less control of B over A. The crucial point here is that the situational context determines behavior to some extent–this is paraphrased as ‘affordance’–i.e., a certain situation tends to propose a certain reaction or activation. In a close, intimate relationship, one partner’s distressed crying suggests a comforting behavior of the other rather than responding to it with aggressive dismissive behavior. However, this example shows that the social dimension of human experience and behavior is more complex.

Therefore, the theory further differentiates that social interaction is a process that is determined by the motivational, emotional, social, instrumental, and potential costs and benefits or outcome of one’s own and the other’s behavior–for example, if someone has to sacrifice something because of the other or can only achieve something because of the other. Interactions are also influenced by comparative processes, i.e., the comparison with expectations regarding the outcome, which are determined by previous experiences. Finally, interaction behavior is determined by habituated patterns of behavior that match with similar situations and additionally follow social norms.

In summary, it is clear that, behavior associated with depression should be understood as an interpersonal process and is not isolated to, for example, biological deficits of an interaction partner. Both partners in an intimate relationship are agents and responders who influence themselves, each other and thus develop a relationship, based on their social interactions, that can be understood as an emergent phenomenon arising from the interplay of the aforementioned factors [36].

To account for associations within couples, actor-partner interdependence models are used to investigate the relationship between depression and relationship satisfaction in more detail in both cross-sectional and longitudinal study designs. It is an approach inspired by the interdependence theory outlined above, taking into account the mutual influence of dyadic data. The actor effect characterizes the impact that an individual’s score on an independent variable A has on one’s own score on a dependent variable B, taking into account the partner’s score on the independent variable A. Compared to that, a partner effect displays the impact that an individual’s score on a predictor A has on the partner’s score on the dependent variable B taking into account the partner’s score on A.

### Actor and partner effects in the context of depression in couples

While evidence for actor effects is large [37], the findings on partner effects seem to be rather heterogeneous: Some studies have found partner effects for both partners [25, 26], while other studies could only partially confirm partner effects [38] or reported no partner effects [29, 39]. Evidence from cross-sectional data predominantly suggests a stronger actor effect of one’s own depression on the self-rated relationship satisfaction than on the partner’s satisfaction [e.g. 40]. Some studies have cross-sectionally investigated the reverse direction from relationship quality to depressive symptoms. Authors found actor and partner associations to be comparable to their primary analysis [25, 26]. Another study could only partially confirm partner effects [41]. A cross-European study found actor effects to be larger than partner effects [42]. Yet, it should be considered that the direction of effects between relationship satisfaction and depression is purely theoretical—especially when cross-sectional data are involved and there is no temporal order through the study design [43], In the few existing longitudinal studies, the following results were evident: Whisman and Uebelacker [27] tested both directions for the connection between marital discord and depression and vice versa over two measurement points and found various actor and partner effects with comparable size for both genders in middle-aged and older couples. In the context of disengagement, it was found that initial depression in husbands is associated with later disengagement and a decrease in relationship satisfaction over time [44].

### How do we measure relationship quality?

Previous studies on actor and partner effects of depressive symptoms and relationship quality have used different instruments to measure relationship quality ranging from dyadic or martial adjustment [29] to relationship quality or satisfaction [45]. The terms can falsely be used interchangeably when the measured construct in fact differ. The extent to which the partners respond in agreement with respect to different situations is interpreted as relationship satisfaction. Further, the Partnership Questionnaire [46] is a widely established instrument in German speaking countries, which asks for the frequencies of relationship behaviors, that are thought to indicate a higher relationship quality when displayed more frequently. The actually shown behavior is an important aspect of relationship quality. Yet, this approach can be perceived as normative. A higher or lower score in certain behaviors might not reflect a higher relationship quality for all couples and it lacks an evaluation of how a certain behavior is perceived. What is more, the Dyadic Adjustment Scale and the Partnership Questionnaire, focus an intra-relationship level of dyadic relationship experiences instead of an evaluative “we”-perspective on the relationship aspects. The Evaluation of Social Systems Scale [47] measures such an overarching evaluation of couple interactions on the system level (e.g. verbal communication or cohesion). Hence, constructs should be differentiated more precisely and the question remains to which extent the way we measure relationship quality impacts the association with depressive symptomatology. Therefore, the associations between depressive symptoms and aspects of relationship quality should be studied comparing a previously applied questionnaire with one that does not make normative assumptions about a good relationship but rather asks for an evaluation on the couple level.

Against the background of considerations, the aim of the present study was to investigate the cross-sectional associations between depression and relationship quality and system quality for both partners in a sample of couples that also allows comparing clinical and subclinical levels of depressive symptoms. Specifically, against the background of the vulnerability-stress-adaptation model [22] we hypothesized that the health impairment in one partner relates to lower relationship quality and system quality in themselves and their partner. This implies testing the following hypotheses:

**H1: In a sample of different-sex couples,** depressive symptoms in women significantly predicts relationship and system quality in both women (actor effect) and men (partner effect).
**H2**: **In a sample of different-sex couples,** depressive symptoms in men significantly predicts relationship and system quality in both men (actor effect) and women (partner effect).

This study was part of a larger project comparing the psychobiological response to an instructed partnership appreciation task between different-sex couples with depressive disorders in women (Cw/DW) and healthy control couples (LDCs) in a cross-sectional design [3]. This study included an intervention in which Cw/DW were randomly assigned to a Cognitively-Based Compassion Training for Couples (CBCT-*fC*) or treatment as usual [48]. Hence, the present study is based on a secondary analysis of data collected in the cross-sectional part of this project.

## Method

### Participants

A total of *N* = 110 different-sex couples took part in this study. All participants were at least 20 years old and in a relationship of at least two years. Couples were assigned to Cw/DW if the female partner was diagnosed with a current major depressive or recurrent depressive disorder assessed via a Structured Clinical Interview (SCID), which was conducted by trained psychologists. Due to the heightened prevalence of depression in women compared to men, and the psychobiological focus of the overall study combined with the need to keep the trial feasible, this study compared different-sex couples with depressive disorders in women and healthy different-sex control couples. Cw/DW were excluded if women or men had psychotic symptoms, a bipolar disorder, acute suicidal tendency or present substance abuse. LDCs were included if they had no current psychiatric diagnosis assessed via a SCID interview [49].

Participants were recruited by advertisement in newspapers, social media, university mailing lists, and on public transport. Additionally, Cw/DW were informed about the study by their psychiatrists, psychotherapists in outpatient or inpatient settings. This study was approved by the Ethics Committee of the Medical Faculty at Heidelberg University (S-021/2016). All participants gave written informed consent prior to participation.

### Procedure

The study was conducted at the Institute of Medical Psychology at Heidelberg University Hospital in Germany. Couples interested in participating were briefly screened in a standardized telephone interview (10 minutes) about their relationship status and duration, current and previous psychiatric disorders and diseases. Cw/DW who could not be included in this study due to the exclusion criteria were informed about alternative treatment options. Couples who met the inclusion criteria were invited to two laboratory assessments on consecutive days. On lab day 1, participants were informed about study goals, procedures, potential risks and benefits. Participants and interviewer signed a consent form. A structured clinical interview for DSM-IV [49] was led to assess any present or past psychiatric disorder. Current depressive symptoms were assessed using the Patient Health Questionnaire-9 (PHQ-9) [50] as a self-report measure. Partners were interviewed consecutively while the other was answering a SoSci Survey tablet questionnaire on demographic and health data (including information on education, income, employment, physical activity, health status, and menstrual cycle) as well as psychometric scales including a measure of relationship quality assessed by the Partnership Questionnaire (PFB) [46] and system quality measured by the Evaluation of Social Systems Questionnaires (EVOS) [47]. On lab day 2, couples were medically screened before they were instructed to participate in a Partnership Appreciation Task [3].

### Measures

#### Patient health questionnaire-9

Depression was measured with the German version of the PHQ-9 consisting of nine items on a four-point scale [40]. In the validation study of the German version of the instrument, composite reliability scores indicated high reliability [40]. In this study, internal consistency was excellent, with α = .91.

#### Evaluation of social systems

The EVOS was applied to measure system quality of couples. The 10-item questionnaire is a self-report questionnaire originally developed and validated in German with excellent psychometric properties α = .94 for the subscale relationship quality and α = .90 for collective efficacy [47]. Sample items are “For me, the way we talk with each other, is…”, or “For me, how we adapt to change, is …,”. In this study internal consistency was α = .91.

#### Partnership questionnaire

The PFB measures relationship quality on the subscales ‘conflict behavior’ with items such as ““When we quarrel he or she keeps taunting me”, ‘tenderness’ with items such as “He or she caresses me tenderly”, and ‘commonality/communication’ with good discriminative and prognostic validity. Internal consistency in a standardization study was excellent, with *α* = .93 [51]. In this study, internal consistency was good, with *α* = .89.

### Statistical analyses

Descriptive analyses for all study variables are presented in Table 1. The degree of non-independence between partners was tested with Pearson’s correlations as dyad members were distinguishable (because couples consisted of persons who identified as women and men). Separate actor partner interdependence models were performed to analyze the association between partners regarding depressive symptoms (independent variable (IV): PHQ-9) and relationship quality (dependent variable (DV): PFB) as well as depressive symptoms (IV: PHQ-9) and system quality (DV: EVOS) (H1, H2). Basically, actor and partner effects are partial correlations, controlling for the other partner’s variable score (e.g., the correlation between women’s depressive symptoms and her partner’s relationship quality controlling for her partner’s depressive symptoms). Using structural equation modeling with lavaan [52], multi-group analyses were performed to address the unbalanced distribution of depressive symptoms. For this approach, Cw/DW and LDCs were compared with a dichotomous grouping variable representing the abovementioned criteria. Firstly, models with variance between actor and partner effects within and between groups were calculated, before testing whether constraining actor and partner effects to be equal within groups or within and between groups fitted the data equally well [53].

**Table 1:** Sample characteristics and descriptives.

			Cw/DW		LDCs	ANCOVA statistics
	Gender	N	M (SD)	N	M (SD)	
**Age (years)**	Women	46	41.97 (13.38)	61	38.74 (16.67)	Gender: *F* = 0.916, *p* = 0.340, ηp^2^ = 0.004; GROUP: *F* = 2.659, *p* = 0.104, ηp^2^ = 0.012; Gender*GROUP: *F* = 0.006, *p* = 0.936, ηp^2^ = 0.000
	Men	45	44.16 (13.61)	60	40.67 (17.54)	
**Relationship (years)**	Women	48	10.09 (11.17)	62	11.42 (12.63)	Gender: *F* = 0.053, *p* = 0.818, ηp^2^ = 0.000; GROUP: *F* = 0.203, *p* = 0.653, ηp^2^ = 0.000; Gender*GROUP: *F* = 0.541, *p* = 0.463, ηp^2^ = 0.002
	Men	48	11.58 (10.44)	62	9.73 (13.19)	
**Disorder (years)**	Women	45	10.81 (10.41)	14	10.07 (12.99)	Gender: *F* = 19.310, *p* = 0.000, ηp^2^ = 0.084; GROUP: *F* = 20.800, *p* = 0.000, ηp^2^ = 0.090; Gender*GROUP: *F* = 16.327, *p* = 0.000, η**p2 = 0.072**
	Men	7	7.52 (11.32)	4	11.88 (12.33)	
**Depressive Symptoms**	Women	48	13.46 (5.86)	61	4.59 (4.66)	Gender: *F* = 31.518, *p* = 0.000, ηp^2^ = 0.129; GROUP: *F* = 51.778, *p* = 0.000, ηp^2^ = 0.196; Gender*GROUP: *F* = 29.712, *p* = 0.000, ηp^2^ = 0.122
	Men	47	5.64 (4.57)	61	3.97 (4.4)	
**Relationship Quality**	Women	47	48.84 (11.28)	62	55.02 (10.67)	Gender: *F* = 0.008, *p* = 0.928, ηp^2^ = 0.000; **GROUP: *F* = 25.133, *p* = 0.000, ηp^2^ = 0.109;** Gender*GROUP: *F* = 0.144, *p* = 0.705, ηp^2^ = .001
	Men	48	49.43 (8.48)	60	56 (9.57)	
**System Quality**	Women	46	27.37 (5.24)	61	31.26 (4.73)	Gender: *F* = 1.083, *p* = 0.299, ηp^2^ = 0.005; **GROUP: *F* = 28.132, *p* = 0.000, ηp^2^ = 0.117;** Gender*GROUP: *F* = 0.541, *p* = 0.463, ηp^2^ = 0.003
	Men	48	28.56 (4.35)	61	31.54 (4.89)	

*Cw/DW* = high depressiveness couples; LDCs = low depressiveness couples; *n* = 62 LDCs and *n* = 48 *Cw/DW*; *n* partially reduced due to missing values; disorder (years) describes duration of any former (remitted) psychological disorder for men in *Cw/DW* and both partners in LDCs; disorder (years) in women in *Cw/DW* describe disorder of depressive disorder that is still present. ANCOVA = analysis of covariance; bold effects were statistically significant on the level of *p* < .05.

## Results

### Baseline characteristics and bivariate correlations

Women in ***Cw/DW*** had significantly higher PHQ-9 scores than women in LDCs (*t* (88.32) = -8.5714, *p* < .001), while men in ***Cw/DW*** had higher but not significantly different PHQ-9 scores compared to men in LDCs (*t* (106) = -1.926, *p* = 0.056). System quality was rated higher in LDCs compared to ***Cw/DW*** in both women (*t* (214) = 5.297, *p* < .001). Similarly, partnership quality was rated higher in LDCs in both partners (*t* (213) = 4.976, *p* < .001). Age and relationship duration did not differ between the groups (p > 05). Descriptive statistics for women and men in both groups are presented in Table 1. The large majority of participants reported a German nationality (93,86% of women and 95.73% of men) and a high school leaving diploma or university degree (74.6% of women and 67.5% of men). Correlations for women, men and between gender were tested with Bonferroni corrected bivariate Pearson correlations. The two relationship measures EVOS and PFB were highly correlated for women (*r* = .77, *p* < .001) and men (*r* = .69, *p* < .001) without being redundant, indicating that measures represent similar, but not fully overlapping, constructs. In women, PHQ-9 was negatively correlated with EVOS (*r* = -.33, *p* < .001) and PFB (*r* = -.23, *p* < .05). In men, correlations between PHQ-9 and EVOS (*r* = -.17, *p* < .05) and PFB (*r* = -.21, *p* < .05) were smaller. Partial correlations between partners controlling for the predictor variable (PHQ-score) indicated a significantly high level of non-independence in system quality (EVOS; *r* = .78, *p* < .001) and relationship quality (PFB; *r* = .64, *p* < .001). Therefore, the dyadic structure of the data should not be ignored. All correlation coefficients including the associations between women and men are shown in Table 2.

**Table 2:** Correlations of study variables.

	EVOS	PFB	PHQ
**EVOS**	.78***^1^	.77***	-.43***
**PFB**	.69***	.64***^a^	-.37***
**PHQ**	-.25**	-.22*	.36***^b^

Pearson correlation coefficients for women above the diagonal; correlations for men below the diagonal;

^a^ Partial correlations between women and men controlling for both of their depressive symptom levels.

^b^Pearson correlation between depressive symptoms of women and men. Correlations are Bonferroni-corrected for multiple testing * *p* < .05; ** *p* < .01; *** *p* < .001.

### Actor partner interdependence models

#### Depressive symptoms and system quality

**Fig 1:**
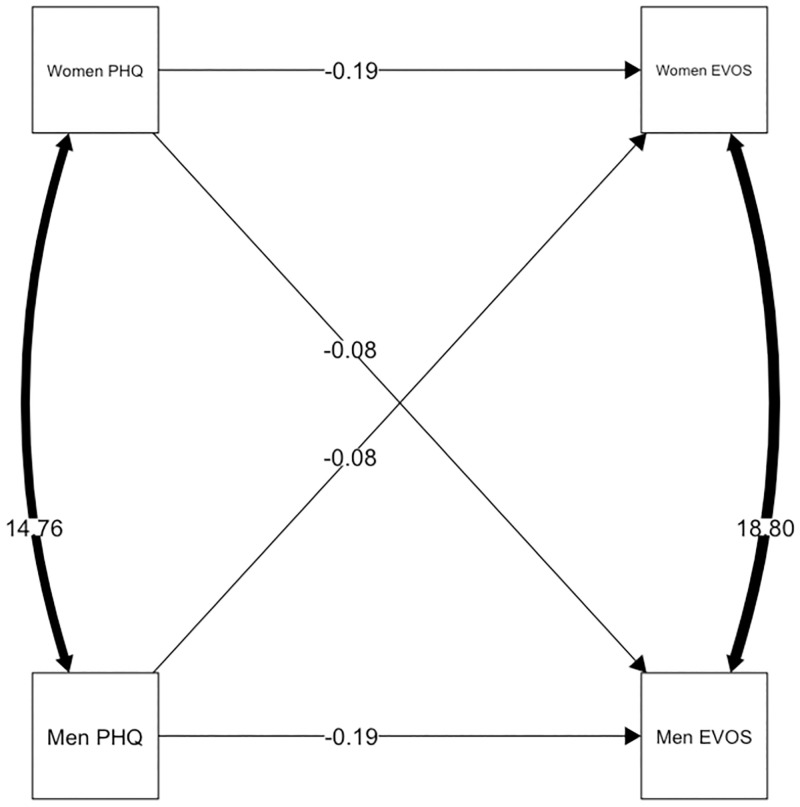
Equal actor and partner effects for system quality and depressive symptoms. Paths constrained to be equal across both gender and group (Cw/DW /LDCs); paths show unstandardized estimates.

Depressive symptoms were negatively associated with one’s own evaluation of system quality in both, women and men and in both, Cw/DWs and LDCs. The overall actor effects were *b* = -0.193 (*p* = 0.008, CI [-0.336; -0.050]). There were no overall significant partner effects of depressive symptoms on system quality (*b* = -0.077 (*p* = 0.264, CI [-0.213; 0.058]). The model fit did not differ significantly from models with different actor and partner effects for women versus men (χ^2^ = 2.606, *p* = 0.272) or one with different effects for Cw/DWs versus LDCs (χ^2^ = 2.606, *p* = 0.272). In both groups, the intercepts for women and men were not significantly different from each other (Δ_HDCs_ = 1.500 *p* = 0.262, 95% CI [CI -1.070; 3.936]; Δ_LDCs_ = -0.071, *p* = 0.666, 95% CI [-2.079; 1.329]), indicating there was no main effect of gender. Concerning the relationship between depressive symptoms and system quality, the model without gender or group as moderator had the best fit (χ^2^ = 36.220 (χ^2^_group one_ = 19.581; χ^2^_group two_ = 16.639). The explained variance in this model in *Cw/DWs* was *R*^*2*^ = .076 for women and *R*^*2*^ = .063 for men. The explained variance in LDCs was *R*^*2*^ = .073 for women and *R*^*2*^ = .051 for men (Fig 1).

#### Depressive symptoms and relationship quality

**Fig 2:**
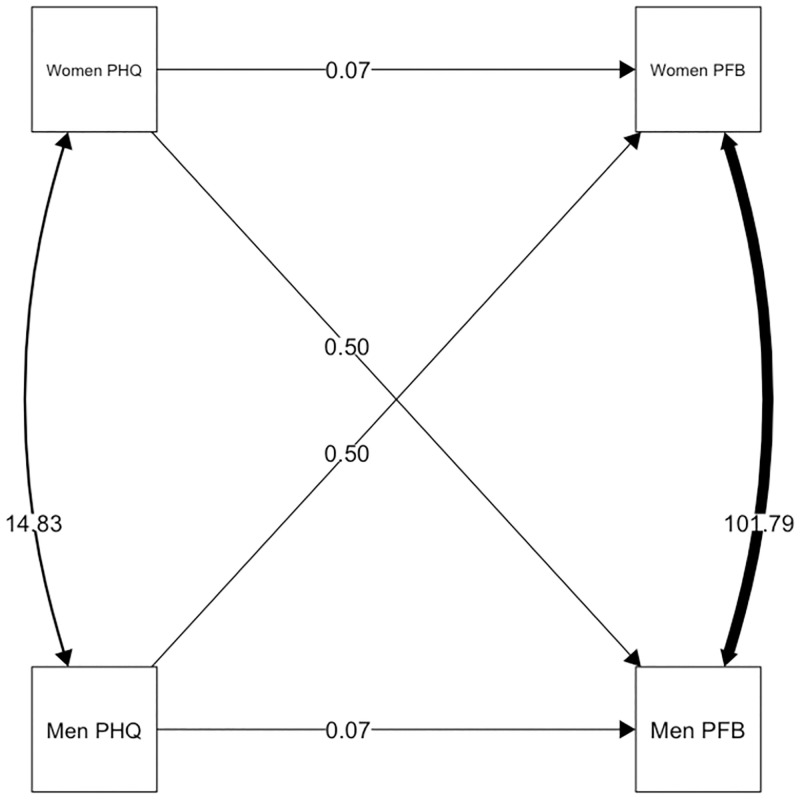
Actor and partner effects for depressive symptoms and relationship quality in LDCs. Equality constrains across genders but not groups; Fig 2 shows LDCs; paths represent unstandardized estimates.

**Fig 3:**
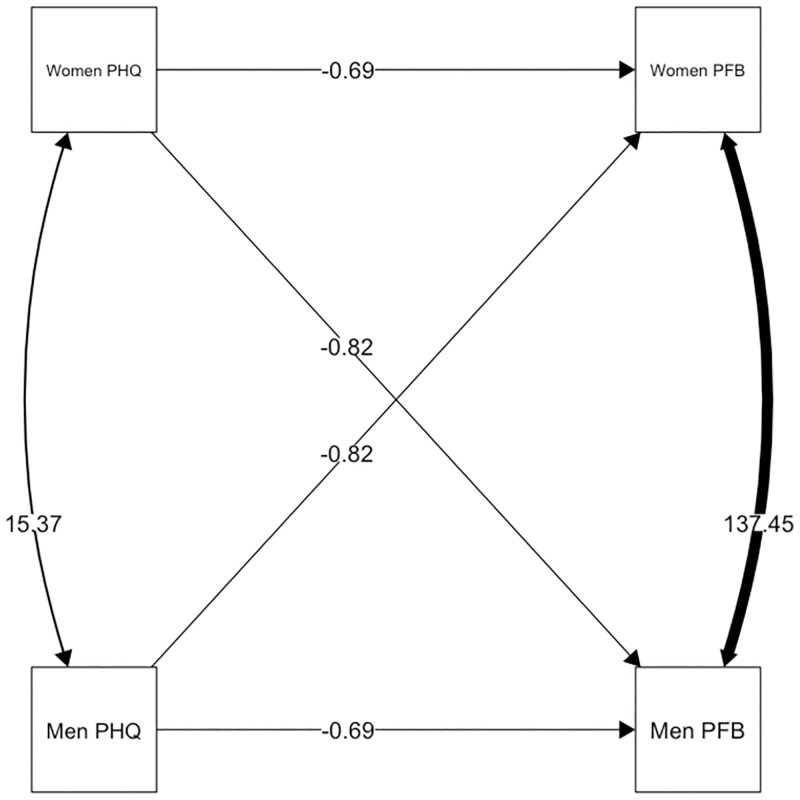
Actor and partner effects for depressive symptoms and relationship quality in Cw/DWs. Equality constrains across genders but not groups; Fig 3 shows Cw/DWs; paths represent unstandardized estimates.

Depressive symptoms were negatively associated with one’s own evaluation of relationship quality in both, women and men in LDCs. The overall actor effects were *b* = -0.688 (*p* = 0.068, CI [-1.425; 0.050]). The overall actor effect in Cw/DWs were marginal and remained insignificant (*b* = 0.067, *p* = 0.816, CI [-0.497; 0.631]). There were significant negative partner effects for both women and men in LDCs of *b* = -0.823 (*p* = 0.030, CI [-1.565; -0.081]). Again, no significant partner effect was observed in Cw/DWs (*b* = 0.499 (*p* = 0.077, CI [-0053; 1.052]). The model fit for this model was better compared to a model with constraints between Cw/DWs and LDCs (χ^2^ = 8.873, *p* = 0.012). Contrarily, a model with gender as moderator did not provide a better fit (χ^2^ = 0.398, *p* = 0.983). The intercepts were not significantly different from each other in LCDs (Δ_intercept_ = -0.382, *p* = 0.907, 95% CI [-6.820; 6.055]) and Cw/DWs (Δ_intercept_ = -3.335, *p* = 0.415, 95% CI [-11.351; 4.681]), meaning that there was no main effect for gender. Regarding the relation between depressive symptoms and relationship quality, the model that constrained actor and partner effects to be equal within groups had the best fit to the data, χ^2^ = 32.638 (χ^2^_group one_ = 18.025, χ^2^_group two_ = 14.613). The explained variance in this model in *Cw/DW* was *R*^*2*^ = .030 for women and *R*^*2*^ = .084 for men. The explained variance in LDCs was *R*^*2*^ = .133 for women and *R*^*2*^ = .162 for men (Figs 2 and 3).

## Discussion

The present study aimed to contribute to a better understanding of the interpersonal impact of depressive symptoms in a sample composed of high and low levels of depressive symptoms. In light of the previously heterogenous operationalizations of relationship quality we compared to different facets. Regarding system quality, we found evidence for equal actor effects in women and men in both high and low levels of depressive symptoms. In our results we found negative actor effects of depressive symptoms on perceived social system quality (EVOS) indicating that the higher the degree of depressive symptoms, the lower the own perceived system quality. Yet, no significant partner effects could be confirmed in both groups regarding system quality. Concerning relationship quality (PFB), actor effects in women and men were marginal and remained insignificant but our results showed negative partner effects on relationship quality in the group of couples with subclinical depressive symptoms, meaning that the higher the depressive symptoms, the lower the partners’ perceived relationship quality. Hence, both hypotheses were only partly confirmed.

Although EVOS and PFB were correlated, different models were preferred. This suggests that the moderating variable of clinically relevant levels of depressive symptoms played a larger role concerning relationship quality than system quality. With regard to the interdependence theory [35] this indicates that in our sample, couples depressive symptoms influenced each other’s relationship quality (partner effects) and their own (actor effects) differently depending on the levels of (clinical) depressive symptoms. In contrast, we can carefully state partners did not significantly differ in their influence on their own and their partner’s system quality. The PFB focuses specifically on behavioral aspects of relationship quality on a “me-you”-perspective, while the EVOS captures a higher-level evaluation of system quality and collective efficacy on a “we”-perspective. The different results between the two questionnaires encourages us to think about issues in operationalizing the relationship quality, functionality or satisfaction. In previous couple research, different constructs such as dyadic adjustment or relationship satisfaction were used to measure the quality of a relationship quality. Researchers have pointed out the difference in measuring an intrapersonal view (individual satisfaction with the relationship) or an interpersonal aspect of the relationship (e.g. dyadic adjustment). Although we expect the constructs to overlap, they are likely to picture different aspects of a relationship [54]. Therefore, we decided to use two different measures. The PFB focuses not only on context-specific aspects of the relationship, but also on concrete relationship behavior, such as “He/She expresses disparaging remarks about an opinion I have expressed” (subscale dispute behavior). In contrast, the EVOS scale focuses not only on cross-context aspects of relationship quality, but also includes items on higher-level dimensions of relationship quality rather than concrete behavior: “For me, the way we talk with each other, is, …”. Answers to this question indicate whether the respondent views the communication positively or negatively, but not communication behaviors such as arguing or silence. Our results mark differences between the questionnaires: On the one hand, different models were preferred: Only regarding the PFB, it was necessary to model variation between *Cw/DW* and LDCs. This could imply that the frequency of adaptive relationship behavior relates differently to depressive symptoms depending on the levels of depressive symptoms, while for the higher-level evaluation of system quality on a “we”-perspective, this distinction is not relevant. Although the generalizability of our results is limited for several facts, the measures may relate differently to depressive symptoms. This underlines the importance of how we choose to measure relationship quality.

Our findings indicate, that gender does not significantly moderate the associations of system and relationship quality with depressive symptoms in subclinical and clinical levels, From the results we can conclude that there were significant negative actor effects of depressive symptoms on system quality regardless of gender and group. Similarly with regard to relationship quality, the significant negative partner effects did not differ between gender. Therefore, our hypotheses on partner effects can partly be supported, depending on the outcome. From our findings we can carefully conclude, that the gender gap in depressive symptoms in our sample seems less relevant when studying depressive symptoms in relation to aspects of relationship quality. Depressive symptoms seem to be a more important moderator than gender with regard to the link to relationship quality (PFB). The experience of depressive symptoms in one partner and the emotional impairment can lead to differences in relationship perceptions compared with the other partner, including increased needs for affiliation and social support [55]. Especially when paired with anxious attachment, women (with depressive symptoms) can experience more distress and insecurity in couple conflict [56, 57]. Both partners can face additional burdens in this situation: she through feeling overburdening by the relationship and he through a perceived lack of support from his partner [58] [for a critical review see 59]. Future studies should compare couples with high depressive symptoms in both partners to couples with few depressive symptoms in both partners.

Although in our cross-sectional approach we cannot infer causal relations, the current theoretical background allows for testing both directions, from depressive symptoms to relationship quality or vice versa. In an explorative approach, we tested the direction of effects, e.g. predicting depressive symptoms by relationship quality. In this analysis, we found no significant actor or partner effects for PFB predicting depressive symptoms. In contrast, we found significant negative actor effects in LDCs for EVOS on depressive symptoms, which partly confirms a number of previous studies, reporting evidence for reciprocal partner effects in both directions [25, 26]. Yet, it should be noted that all others paths could not confirm this reciprocity. Testing both directions of effects in a longitudinal design would advance our understanding of the possibly circular association of relationship quality and depressive symptoms.

All together, we can state that controlling for the specific sample composition, including clinically meaningful depressive symptoms in half of the women, our results include actor and partner effects reflecting the severe and detrimental consequences of mental illness on both relationship partners, providing more support for the hypothesis of depression as a classical “we-disease” [60]. The partner effect on relationship quality in LDCs points to the importance to make the relationship a discussion even when partner experience low levels of depressive symptoms as compared to a full pronounced depressive disorder. Preventive settings that include both partners could help to inhibit a vicious circle of negative effects between depressive symptoms and relationship quality.

### Strengths of the study

A strength of our study is the sample that allowed comparing actor and partner effects in clinical and subclinical levels of depressive symptoms in couples. This is to our knowledge the first study to do so. In addition, we compared to ways to measure the quality of relationship, which represent different levels of evaluation: an insight-level measure of relationship quality focusing on behavioral aspects and a higher-level evaluation of system quality and collective efficacy. With this approach, we aimed to examine whether couple-specific relationship quality with a focus on behavioral aspects alone play a role in the connection between depressive symptoms and relationship quality. This is important because, on the one hand, (sexual) intimacy or passion are often emphasized in romantic relationships and thus also in relationship models (aspects which mark a difference to other social systems). On the other hand, in the case of depressed patients, the decline in sexual intimacy and emotional responsiveness is particularly emphasized [61]. The different APIM results between the two questionnaires used in our study underlines the importance to carefully operationalizing relationship quality characteristics. Interpersonal aspects that play an important role for different social systems (not only for couples), such as communication, cohesion and adaptability, are crucial. Although in the context of couple research the prediction of hostility and conflict is important in the course of the relationship and couple therapy, a non-judgmental (non-deficit-oriented) and non-normative assessment of what constitutes relationship quality and collective efficacy (which was possible with the EVOS questionnaire) is highly relevant.

### Limitations

Our study had several limitations that should be considered when interpreting the findings. A major limitation of this study is the cross-sectional design, which does not allow for an interpretation of the causality of effects. It is up to future studies to examine the reciprocal and longitudinal effects of different operationalizations of relationship quality and depressive symptoms. This would also allow to test effects from marital dissatisfaction on depressive symptoms comparing both operationalizations with regard to Beach’s marital discord model [10, 11]. Further, reciprocal or circular effects could be studied. Furthermore, we included a sample of different-sex couples, educated as well as socio-economically well-situated, which limits the generalizability of our results. On the one hand, our sample can be viewed as highly specific comparing couples with women either having depressive disorders or not, while their male partner reported depressive symptoms in the non-pathological range. From this perspective, this represents a limitation of our study. On the other hand, this reflects the up to this day uneven distribution of depressive disorders in the general public [15]. Future research should try to include a more diverse sample with regard to race, gender identity, sexual orientation, education, socio-economic background and also include couples with depressive disorders in men. These aspects might influence the results and furthermore, reflects the reality of diverse lives.

### Future directions

Future research should test other potential moderators and mediators of the association between depressive symptoms and relationship quality in longitudinal study designs. For example, social competence and interpersonal skills are important potential moderating or mediating factors, e.g. following the skills-based model of healthy relationship functioning: Mentalizing and self-reflection skills (e.g. a realistic self-assessment), the ability to self-care, to empathize and take perspectives with regard to one’s own needs and those of others, up to (co-)regulatory skills in dealing with the whole range of emotionally tinged situations and stress-related life circumstances. Common factors impacting depressive symptoms as well as the social relationship, such as stressful events or contextual factors, should be included in studies, since depression has been associated with deficits in individual and dyadic coping in couples [15]. Future studies should clarify the reciprocity of this linkage more precisely and to take into account gender, type and duration of the relationship and other demographic variables that may influence it–aspects that have hardly been taken into consideration so far. Moreover, future studies should take a closer look at different types of romantic relationships. Focusing not only on married, different-sex couples, but also on very different relationship scenarios (same sex couples, different gender identities, etc.) will help to better address the plurality of life practices in our society. The same applies to the variety of demographic and socio-economic characteristics.

## Conclusion

Our findings support the important notion of the interpersonal nature of individual distress and its social contextual embeddedness. Our results include actor and, controlling for clinically relevant depressive symptoms, partner effects on system and relationship quality. In the scope of clinical interventions, it is therefore important to consider the partner in the context of individual psychotherapy: Not only to reduce the individual symptoms and to influence the course of the relationship in a positive way, but also to minimize the burden imposed on the partner. In this context, the implementation of specific interpersonal aspects could be of importance when it comes to forming and utilizing the relationship as a resource for health and well-being.
